# The study of optimal nursing position in health care delivery system in Iran

**Published:** 2010

**Authors:** Maryam Sadat Shahshahani, Shayesteh Salehi, Mohammad Rastegari, Abdollah Rezayi

**Affiliations:** * Nursing Education, Department of Nursing Health, School of Nursing and Midwifery, Kashan University of Medical Sciences, Kashan, Iran; ** Associate Professor of Nursing Education, School of Nursing and Midwifery, Islamic Azad University, Khorasgan Branch, Isfahan, Iran; *** Nursing Education, Department of Nursing Health, School of Nursing and Midwifery, Isfahan University of Medical Sciences, Isfahan, Iran

**Keywords:** Nursing, position, description, health care systems

## Abstract

**BACKGROUND:**

In the recent decade, due to the overwhelming importance of health and prevention of diseases, nurses, the greatest part of the health care system, are acting in any position of the health care delivery system; because nursing have a key role in promotion of health and health care everywhere. The objective of this research was to study the desired positions of nursing in the health care delivery system in Iran.

**METHODS:**

This was a triangulation study done on three steps during 2005-2007. At the first step, the positions of nurses were elicited out of library and internet sources. At the second step, the comments of 15 participants were collected using an open questionnaire. Thereafter, at the third step, using the collected data, a questionnaire was made for a poll (all over the country) on the optimal positions of nursing in Iran, and 64 participants answered it. The results were analyzed using descriptive statistics.

**RESULTS:**

Derived positions were categorized in two groups: hospital, and community positions. The results showed that all positions were accepted more than 70%.

**CONCLUSIONS:**

Considering positions of nursing and in order to promote the nursing itself and community health, it is suggested that proper planning should be implemented for nurses’ activities in these positions by health planners.

In recent decades, health has been considered as one of the human rights. Health is a social objective and a large social investment all over the world.^1^ Today, the most important concern of mankind and health care delivery system is promoting and protecting health in many aspects through policy, inner sovereignty and inner leadership.^2^ Each government has been in charge of providing available health care for citizens and also proper usage of health and medical facilities.^3^

Currently, there has been created tremendous changes in medical and health care delivery systems all over the world.^4^ Reduced hospitalization time, increased outpatient surgeries, emphasizing on the health improvement, identifying the health requirements of patients, and cost reduction tendencies have made some changes in health care delivery system.^5^ These changes have given more attention to the communitybased cares, and considered hospitalization only for emergency cases.^6^ In an article published in early 1990s, the United States Institute of Nursing has pointed out moving from health and care- which are the most expensive levels of care- to the primary care-based level; for example, in the United States, 5% of urban population allocates 58% of health budget, while 75% of this budget has spent for the health and hospital services.^6^

Conger et al believed that reformed movement of health care delivery system is toward community-based systems direction. They also believed that hospitals and clinical centers should be considered as a part of health care delivery system not as health services centers.^7^ Thus, in the past decade, the position of health care delivery system also has been affected by the severe and significant changes.^8^

Today, health care system in Iran is implemented as a system. In recent years, health care delivery system, which has taken the responsibility of providing health and treatment of all the citizens, had focused on the primary health care approach as the base of the new design for service system. This system has emphasized on the preventive and outpatient cares instead of hospitalization services.^9^ These basic changes on the health care delivery system have had a direct effect on health careers, and consequently, it changed the role of employed experts in the system.^10^ Considering the fact that most part of the human forces of this system includes nurses, consequently it has some effects on nursing.^6^ Nowadays, principles of health care delivery system are based on recognizing the patient, individual and community health.^11^ In nearly past decade, there has been created a certain change in location and nature of nursing cares from hospitals to the primary and community-oriented cares.^12^

In 1999, the United States Institute of Nursing proclaimed that during the past decade, nursing discipline has emphasized on increasing importance of health improvement and illness prevention in any place in the health care delivery system. Moreover, many institutes such as the United States Institute of Nursing have come to realize that homes, workplaces, schools, churches and local clinics are proper places as “appropriate and familiar places” for increasing principled and comprehensive preventive care services.^5^ In fact, all levels of health and care have led toward community-based nursing.^5^

The data indicate the largest change in nursing toward increasing employment in community-based practice.^6^ In all the environments, nursing has a key role in health promotion.^13^

In Iran, in spite of the fact that manpower classification in the system must be 1 family physician and 0.69 nurse or auxiliary nurse per 2626 people for receiving health services,^14^ but unfortunately the position of this career is unclear^15^ and a few studies have been accomplished about the nursing.^15^–^17^ In a review, positions of countries such as the United States (North Carolina, Tennessee), the United Kingdom, Australia, India, Singapore, Canada and etc were evaluated. Nursing career must try to indicate its potential capabilities by recognizing the position they are in.^18^

Based on the knowledge of the health care delivery system and also the capabilities of nursing which can meet the needs of individual, family and the society, the researcher decided to assess the optimal position of nursing in Iranian health care delivery system considering the new approach of health and treatment services system.

## Methods

This study was done between 2005 and 2007 in multiple triangulation method. The methods of Delphi and survey were used.

At the first step, the positions of nursing have been elicited out of library and internet sources. At the second step, the comments of participants have been collected by means of an open questionnaire. This made happen by comments of 15 experts who were faculty members of Nursing and Midwifery Schools, MSc and PhD nursing students and also nurses of Isfahan. Thereafter, at the third step, using the data collected in the first and second steps, a questionnaire was made up for a poll (all over the country) on the optimal positions of nursing in Iran, and 64 participants replied; considering that people must be chosen with knowledge of the research subject, it was a purposive sampling. Therefore, they were asked to present the existence and ideality of functions. Finally, the nursing position in medical and health services system in Iran was presented. At the second and third stages, nurses with formal or contract employment in health care delivery system, working in affiliated centers of medical universities, in BS or MSc degree with maximum and minimum of 15 and 5 years of experience respectively or the nurses who were faculty members of Nursing and Midwifery Schools with at least MSc degree or managers of health care delivery systems (training supervisors, matrons and all the members of different fields in management position of health care delivery system) or students of MSc and PhD in nursing were enrolled.

Finally, the results were analyzed using descriptive statistics.

## Results

The results showed that all positions were accepted by 70% and more. Many different positions have been achieved from the library and internet resources such as: Taylor et al,^19^ North Carolina Nurses Association,^20^ North Carolina Center for Nursing,^21^ Nurses for a Healthier Tomorrow,^22^ Cherry and Jacob,^18^ Nursing in Tennessee,^23^ Mayo School of Health Sciences,^24^ American Association of Colleges of Nursing,^25^ United Kingdom’s National Health Services Career,^26^ Daly and Carnwell,^27^ Krisp and Taylor,^11^ Singapore Health Professionals Portal,^28^ Craven and Hirnle,^29^ Canadian Nurses Association (Nursing in Canada),^30^ Indian University’s School of Nursing (Bloomington).^31^

Some of the positions have been made in the second step from viewpoints of experts. Finally, the positions were categorized in 2 groups: hospital and community positions. The hospital positions included all the hospital wards (heart, lung, gastrointestinal, orthopedic, operating room, emergency department patients dying and etc), tubing sections (angiography, endoscopy and etc), and infection control center and telemetry units (medical care through advanced communications equipment and facilities).

Community positions were as follow:

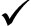
 Fire-fighting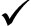 Army/Armed forces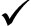 Camping/Refugees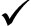 Travel agency/Airplane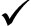 Hospice/Nursing home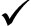 Ambulance/Helicopter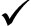 Special care organizations and associations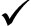 Nursing associations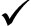 Club/Coliseum/Saloon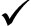 Blood transfusion base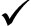 Providing health care at home institutions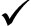 Nursing faculty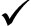 General media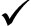 Prisons, Detentions/House of correction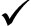 Network health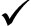 Rural home health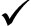 Rural health centers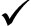 City health base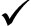 City health centers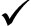 Drug producers and medical supplies factories and companies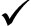 Insurance company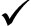 Municipalities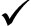 Primary care clinics (families, mothers, infants and…)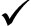 Working environments (offices, factories)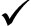 Kindergartens/Preschools/Schools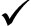 Private nursing center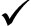 Centers for providing solutions via phone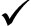 Centers of health plans and education for patients with special needs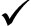 Survey and screening of diseases centers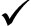 Faculty and university health centers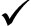 Mobile health centers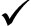 Community health centers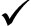 Research centers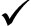 Cessation drugs and alcohol centers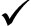 Rehabilitation centers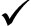 Community services centers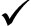 Behavior therapy and psychotherapy centers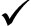 Critical care centers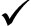 Religious centers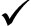 Day care/Outpatient care centers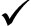 Assistance centers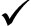 Poisoned centers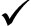 Public consultation centers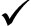 Nursing consultation centers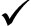 Doctor’s office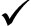 Long-term care provider institutions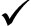 Advanced care/Professional care centers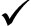 Survey, screening and education units of sexually transmitted diseases

## Discussion

Regarding the results that all the hospital and community positions have been confirmed by the international nursing positions, there are many positions for nursing; while, according to the statistics of ministry of health, almost all the nurses are working in medical and hospital positions.^9^ Lack of knowledge of many nursing capabilities in our country -despite the necessity of all the positions- might caused the low presence of nurses in the community positions. This could also be due to economically saving of many financial costs, promoting the medical and health level of the community, and also shortage of first grade prevention programs in Iran and confining the nurses in the hospitals. If any cooperation and connection happens between the health team members, and any extensive research be implemented in connection with the impact of nurses on community to provide an information for policy makers in order to plan properly, there will be a possibility for providing nursing position which is optimal and desirable.

This research was only a small effort in nursing career. More researches and studies in long term planning of medical and health services are needed to gain quality care improvement, decreasing the costs and also promoting the nursing career. Our findings may be helpful in different areas of nursing, education, research, health and economics.

The authors declare no conflict of interest in this study.
